# Performance evaluation of publish-subscribe systems in IoT using energy-efficient and context-aware secure messages

**DOI:** 10.1186/s13677-022-00278-6

**Published:** 2022-01-31

**Authors:** Norisvaldo Ferraz Junior, Anderson A.A. Silva, Adilson E. Guelfi, Sergio T. Kofuji

**Affiliations:** 1 Laboratório de Sistemas Integráveis, Polytechnic School of Universidade de São Paulo (USP), São Paulo/SP, Brazil; 2 Instituto de Pesquisas Tecnológicas do Estado de São Paulo, São Paulo/SP, Brazil; 3 Centro Universitário SENAC, São Paulo/SP, Brazil; 4 Universidade Paulista (UNIP), São Paulo/SP, Brazil; 5 Universidade do Oeste Paulista, São Paulo/SP, Brazil

**Keywords:** IoT, WSN, Publish-subscribe, Context-aware messages, Security

## Abstract

**Background:**

The Internet of Things (IoT) enables the development of innovative applications in various domains such as healthcare, transportation, and Industry 4.0. Publish-subscribe systems enable IoT devices to communicate with the cloud platform. However, IoT applications need context-aware messages to translate the data into contextual information, allowing the applications to act cognitively. Besides, end-to-end security of publish-subscribe messages on both ends (devices and cloud) is essential. However, achieving security on constrained IoT devices with memory, payload, and energy restrictions is a challenge.

**Contribution:**

Messages in IoT need to achieve both energy efficiency and secure delivery. Thus, the main contribution of this paper refers to a performance evaluation of a message structure that standardizes the publish-subscribe topic and payload used by the cloud platform and the IoT devices. We also propose a standardization for the topic and payload for publish-subscribe systems.

**Conclusion:**

The messages promote energy efficiency, enabling ultra-low-power and high-capacity devices and reducing the bytes transmitted in the IoT domain. The performance evaluation demonstrates that publish-subscribe systems (namely, AMQP, DDS, and MQTT) can use our proposed energy-efficient message structure on IoT. Additionally, the message system provides end-to-end confidentiality, integrity, and authenticity between IoT devices and the cloud platform.

## Introduction

The number of Internet of Things (IoT) devices connected to the Internet continually increases as well as the data produced by these devices [1–3]. The IoT devices provide the services of sensing, monitoring, and automation of activities [4].

Continuous sensory data gathering is essential to support the IoT applications, where devices periodically sense their environment and send the data to the cloud [5]. However, the energy consumption must be in focus [6, 7], either concerning devices’ battery life duration or the energy efficiency of the cloud platform.

Thus, standardized end-to-end messages between the cloud platform and the IoT devices are essential, whether for the cloud to send commands or for the devices to send their measurements [8, 9].

In this scenario, to transmit the IoT application data, various Machine-to-Machine (M2M) protocols can be used according to [10–15]: Constrained Application Protocol (CoAP), Message Queue Telemetry Transport (MQTT), eXtensible Messaging and Presence Protocol (XMPP), Advanced Message Queuing Protocol (AMQP), and Data Distribution Service (DDS). CoAP is a request/response RESTful protocol derived from the Hypertext Transfer Protocol (HTTP) and is designed for constrained devices [10, 15]. CoAP-enabled devices require a request to send data to the cloud, for example. However, when cloud-connected, an important feature of devices is to transmit data without waiting for a request. Therefore, the publish-subscribe systems (XMPP, MQTT, AMQP, and DDS) provide the ability to periodically send data to the cloud [12–14] without the need for receiving a request. Publish-subscribe systems connect endpoints or clients (IoT devices and the cloud platform, for instance) using a Broker.

### Problem fundamentals and contribution

Publish-subscribe systems ensure data delivery to the cloud using TCP as transport and provide two resources to the messages: the topic (a hierarchical list indicating which context the data sent refers to) and the payload.

IoT applications’ data must contain some meaningful context to facilitate interoperability and to help in reducing the processing effort of cloud applications [16–18]. The context must be present to provide metadata (identification of devices and their sensors, deployment location) to the IoT applications’ data.

The context-aware requirement attests to issues related to publish-subscribe systems: the absent standardization of the topic and payload. Although the unstructured nature provides flexibility, it also has drawbacks: topic and payload vary from tens to hundreds of bytes, as observed in the works of [12, 19–21].

Two categories of devices can use publish-subscribe systems: constrained (ultra-low-power) and unconstrained (high capacity). Both enable remote sensing capabilities delivering IoT data to the cloud platform. The ultra-low-power IoT devices are resource-constrained in processing (16 MHz MCU, for example, once are microcontroller-based), memory (∼128KB ROM/Flash, ∼20KB RAM), battery (3V), and payload (∼127 to 256 bytes depending on the protocol) [10]. For this reason, these devices require specific link-layer protocols, such as LoRa and SigFox - for Low-Power Wide Area Network (LPWAN) or Wireless Sensor Networks (WSN) - the latter mainly composed by Low-Power Wireless Personal Area Network (LoWPAN). Conversely, the unconstrained devices commonly utilize (Wireless) Local Area Network (W)LAN, and they are robust enough to use any protocol, whether for messages or security. However, if battery-powered, the energy only lasts a few hours, as observed by [10, 19].

Regarding security between the publish-subscribe clients and the Broker, the Transport Layer Security (TLS) provides robust security, and it is the primary option. However, considering the publish-subscribe systems’ architecture, even the Transport Layer Security (TLS) is not suitable to provide end-to-end security once the IoT data is secure only between the client and the Broker and not between the clients. We observe in the literary works the use of more than one security control [22].

Therefore, the publish-subscribe systems need a message structure to provide end-to-end security because, if the payload does not have security, the Broker can read the messages. Additionally, only the unconstrained devices can use TLS, as observed in [23]. The use of TLS is not an option for ultra-low-power IoT devices given its constrained nature [24, 25]. Nonetheless, even without TLS, the ultra-low-power devices must transmit end-to-end secure publish-subscribe messages, using a security mechanism on the message. In this regard, LPWAN devices do not provide end-to-end security once they need a gateway to connect the devices to the Internet (and consequently to the cloud). LPWAN devices also only send raw data to the gateway and do not enable the devices to use any publish-subscribe protocol. LoWPAN devices, in turn, are natively connected to the Internet (using 6LoWPAN) and can use the publish-subscribe systems to transmit data to the cloud.

Thus, the main contributions of this paper can be summarized as:

- An energy-efficient, context-aware, and end-to-end secure publish-subscribe messages (LWPubSub) for cloud-connected devices;
- An extensive evaluation of the message system using both ultra-low-power devices and high-capacity devices;
- A resource comparison (focused on energy consumption) among devices that uses publish-subscribe systems;
- A comparison of message size among publish-subscribe systems for high capacity and ultra-low-power devices;
- A comparison between the main publish-subscribe systems (AMQP, DDS, and MQTT) when communicating between heterogeneous devices (high-capacity and ultra-low-power) and the cloud platform.

**Fig. 1:**
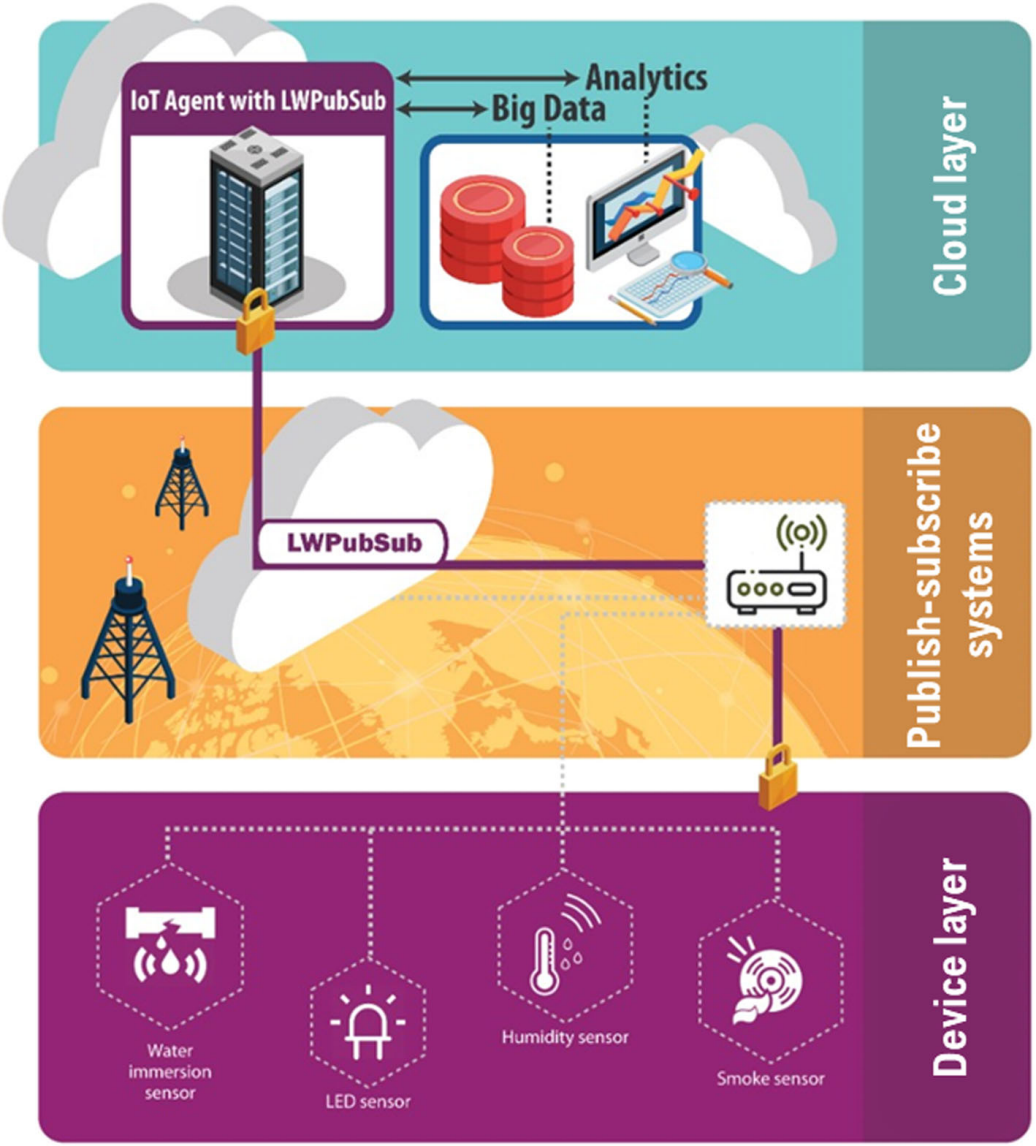
LWPubSub context-aware and secure message using publish-subscribe systems

Figure 1 presents the overview of the use of IoT devices and the interconnection to the cloud using the LWPubSub message structure on publish-subscribe systems.

### Article organization

The remainder of this paper is organized according to the following structure. “Related work” section introduces publish-subscribe systems for IoT, focusing on the necessity of standardization of the messages, reviewing and discussing relevant techniques. “System model” section presents the proposed structure of context-aware and secure messages for publish-subscribe systems. “System evaluation and experiments” section presents the experimental scenario and the parameters for evaluating our proposed message system. “Results and discussion” section presents the results for both ultra-low-power and high capacity devices. Finally, “Conclusion” section provides the conclusion and future work suggestions.

## Related work

This section reviews the related work regarding publish-subscribe systems for IoT applications to clarify our contributions.

### IoT applications’ devices

Remote sensing provides sensing and actuation capabilities to IoT devices. These tasks involve, for instance, measuring environmental conditions (inside/outside temperature and humidity, smoke presence, gases, among others) and health information (for wearables) [3, 28].

The devices send these data to the cloud platform. The LPWAN and LoWPAN devices provide lower energy consumption when compared to unconstrained devices such as Single Board Computers (SBC) [19–21]. In Table 1, we compare the functions and capabilities for remote sensing regarding constrained and unconstrained devices. For LoWPAN, 6LoWPAN is the main protocol for routing packets to the Internet.

**Table 1:** Overview of the main requirements of platforms of IoT devices

**Constrained IoT devices**					
Platform	Battery lifetime [10, 26]	Data rate [26, 27]	Range [26, 27]	Payload [26, 27]	End-to-end to the cloud
LoRa	Years	50 kbps	20 km	243 bytes	No
SigFox	Years	100 bps	20 km	12 bytes	No
6LoWPAN	Months	250 kbps	100 m	127 bytes	Yes
**Unconstrained IoT devices**					
Platform	Battery lifetime [19, 20]	Data rate	Range	Payload	End-to-end to the cloud
SBC	Hours	>54 Mbps (WLAN)	100 m	2312 bytes	Yes

**Fig. 2:**
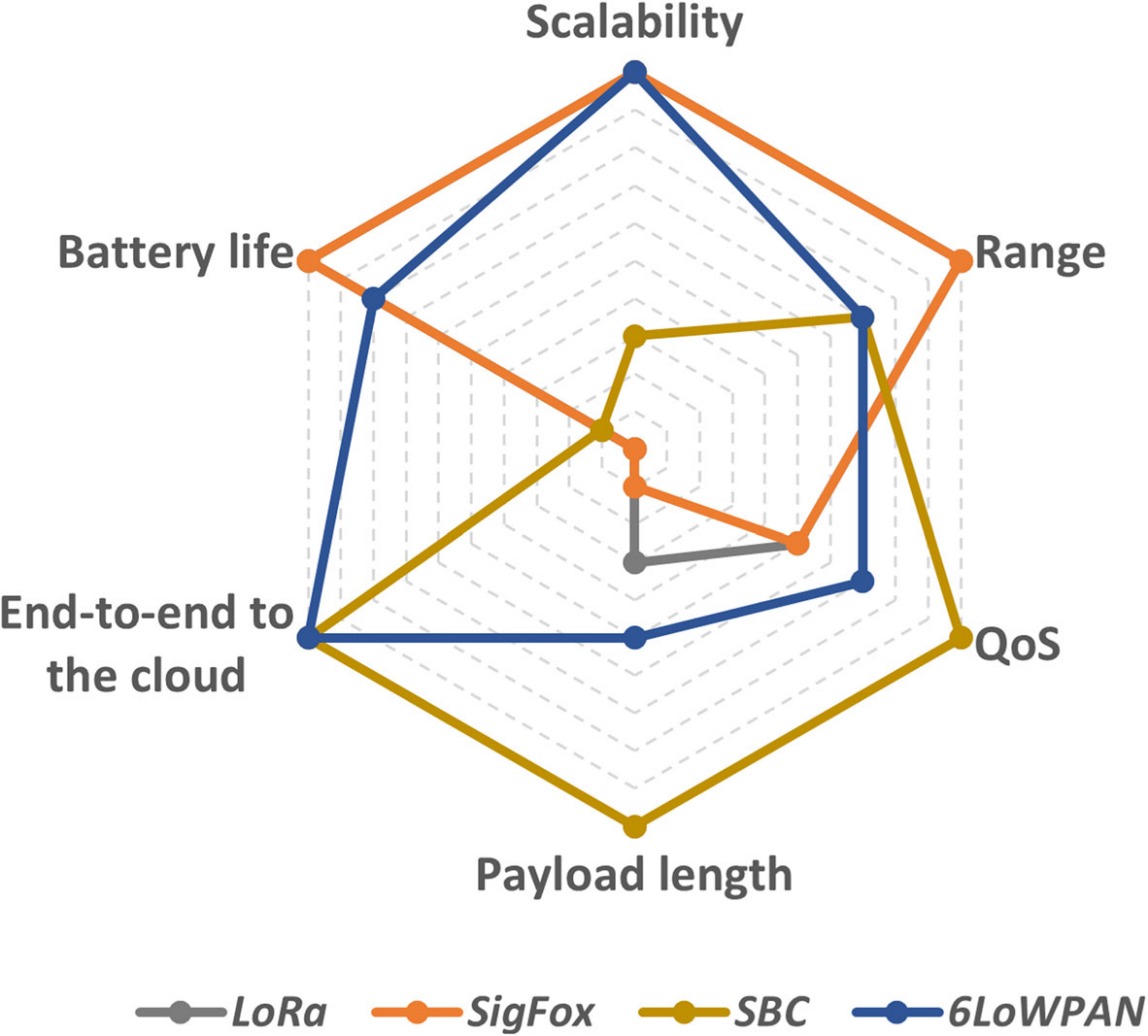
IoT device’s platform comparison regarding battery life, end-to-end data transmission to the cloud, payload length, QoS, range, and scalability

In Fig. 2, we present a graphic representation comparison of the IoT devices presented in Table 1. While being the most energy-efficient, we observe that LoRa and SigFox devices do not provide end-to-end connection to the cloud. In turn, SBC and 6LoWPAN devices provide end-to-end data transmission to the cloud. 6LoWPAN devices have an advantage over battery life, which lasts for months compared to hours of SBC devices.

The 6LoWPAN devices are embedded systems restricted in processing, memory, payload, and energy resources [29]. The connection to the Internet (as stated in RFC 6282) requires the use of the 6LoWPAN Border Router (6LBR) [30]. The 6LBR performs IPv6 packet routing - using the IPv6 Routing Protocol for Low-Power and Lossy Networks (RPL - RFC 6550) - with compressed headers on IEEE 802.15.4 networks (MTU 127 bytes) - the 6LBR is more robust equipment when compared to other nodes in the network.

SBC is microprocessor-based, and this type of device can run general-purpose Operating Systems (OS), such as Linux. Thus, SBC is the leading platform used to evaluate IoT frameworks and message systems, as observed in [19–21, 31].

### Message standardization

In publish-subscribe systems, the topic refers to the context of the payload, and both need standardization to enable interoperability and the correct processing of data. The works of [32, 33] proposes standardization for the topic, using different approaches, but none of the presented works are concerned with interoperability; in contrast, the goal of their work is only to present a specific format.

Tantitharanukul et al. [33] presents the difficulty of a subscriber understanding non-standardized topics for open smart cities. Therefore, the topic structure proposed by [33] requires, at least, the fields “*Objective/Location/Owner*” and other sub-fields.

Vrettos et al. [32], in turn, proposes topics concerning “*networkName/ nodeID/ country/ districtState/ cityTown/ areaDescription/ area/ building/ room/ control*” - a too large topic for remote sensing by ultra-low-power devices. Both works expect clients with direct access to the Broker.

### Publish-subscribe systems

The XMPP, DDS, MQTT, and AMQP are the main protocols used by publish-subscribe systems [10, 11, 13, 14, 34]. These systems are not suitable to discover services to configure the IoT devices automatically [21], and the cloud platform plays an important role at the orchestration and provisioning of the devices which use these message system.

Publish-subscribe systems enable the interaction of multiple endpoints (Broker, publisher, and subscriber) [19, 21]. Interactions between clients are moderated by the Broker, so clients do not need to know each other to exchange messages [21]. A client is a peer, an application, or a device that exchanges application messages about a given topic with another client. The payload is topic-dependent and does not follow any standard. As presented by [35, 36] it is common to use JSON at the payload-agnostic publish-subscribe systems.

XMPP is a communication protocol based on XML, which supports multiple patterns, including asynchronous messaging and publish-subscribe. Therefore, according to [25], data communication requires optimized Internet application protocols. XMPP has its primary use as Instant Messaging (IM), though it also can be used to deliver data between devices and the cloud. However, as presented by [37], XMPP is not originally designed for IoT applications, and it cannot be deployed in its current form on constrained devices. Besides, XMPP does not support acknowledged communication. The main obstacle regarding XMPP is the use of XML language, which causes longer messages, therefore, consuming a larger bandwidth [14].

MQTT is a publish-subscribe messaging protocol and is suitable for devices with resource constraints and networks with low bandwidth and high latency [15]. Its simplicity and small header size compared to other protocols (XMPP, AMQP, and DDS) make it one of the most important options for IoT applications [14]. MQTT defines three Quality-of-Service (QoS) levels for the messages: 0 (at-most-once), 1 (at-least-once), and 2 (exactly-once). This flexible approach assigns complexity to the Broker, resulting in a lightweight header and a small code footprint [38]. MQTT was also designed for asynchronous communications, where subscriptions or publishing from different entities take place in a parallel order [39]. Similar to XMPP, the MQTT protocol uses TCP as a transport. Client communication occurs by the Broker’s request to subscribe or publish messages, specifying the topic and payload fields of the message. Thus, clients, who want to receive a message, subscribe to a specific topic with the message delivered on the payload [3, 14, 21]. Topics are hierarchically organized in a tree structure using the “/” separator.

AMQP (such as MQTT) is another ISO/IEC messaging protocol, developed initially for banking services [34, 38]. Its main purpose is to manage the queues receiving a large number of transactions and to deliver them reliably later. Message queues are queues where messages are routed to and keep them there until the corresponding subscriber reads them. The current version of AMQP enables peer-to-peer communication, not only client-broker communication. However, AMQP requires the higher header amongst the other publish-subscribe systems, and its use for IoT applications is mainly restricted to servers or high capacity equipment [14].

DDS enables high-performance M2M communication using the publish-subscribe paradigm [15]. DDS is a standard presented by the Object Management Group (OMG) and is brokerless [40]. Therefore, DDS is decentralized, and publishers and subscribers exchange data directly between them. Thus, a publisher client publishes data even if there is no subscriber since publishers do not know who uses their data [14]. The DDS defines four main entities: the “Domains” (a virtual entity that allows communication of devices with the same interests), the “Publisher” with its “Data Writer” (used by the publisher to send the data), the “Subscriber” and its “Data Reader” (controlled by the subscriber to read the data), and the “Topic”. In contrast to the previous protocols, DDS supports UDP and TCP, and one of its benefits is the wide range (23) of available QoS [14]. Moreover, the DDS header requires at least 56 bytes, and even the DDS for eXtremely Resource Constrained Environments (DDS-XRCE) [41], require at least 12 Bytes - 8 bytes at the header plus 4 bytes for each *submessageHeader*. For that reason, the works of [10, 40] conclude that DDS consumes at least two times more bandwidth compared with MQTT.

Table 2 summarizes the key features of the publish-subscribe message systems under consideration based on the corresponding literature.

**Table 2:** Key features of the publish-subscribe message systems under consideration

	XMPP	MQTT	AMQP	DDS
Transport protocol	TCP	TCP	TCP	TCP/UDP
QoS support	No	3 levels	3 levels	23 levels
Header size (at least)	Variable (XML tags)	2	8	12*
Security	TLS	TLS	TLS	TLS/DTLS
Encoding format	XML	Binary	Binary	Binary
Low-Power and Lossy networks	Fair	Good	Good	Poor
Standard	IETF	OASIS	OASIS	OMG

*DDS-XRCE

We conclude, regarding the considered publish-subscribe systems, that the following protocols produce larger packets (from the largest to the smallest): XMPP, DDS, AMQP, and MQTT.

Regarding security, high-capacity IoT devices, such as SBC, can use standard protocol (TLS, for example). However, ultra-low-power devices are not capable of using TLS. Consequently, a security mechanism must be in place to protect the data regardless of the used publish-subscribe system because the Broker is an intermediary in this communication. Thus, if the payload does not have security, the Broker can read the messages. Even using TLS, the payload needs security not to reveal data to entities other than the clients.

Thus, we propose a structure for the topic and payload for publish-subscribe systems that can benefit either constrained or high capacity IoT devices (for those capable of sending end-to-end messages to the Internet without proxies). The innovation in our message structure is that the proposed structure for topic and payload is energy-efficient and secure. The energy efficiency is twofold: for devices, the message improves battery lifetime, and for cloud platforms, it reduces network traffic and power consumption. Besides, the messages are end-to-end secure between devices and the cloud platform once only TLS cannot deal with end-to-end security between devices and cloud, considering the Broker’s presence that forwards the publish-subscribe messages.

## System model

This section presents our LWPubSub message structure, providing secure and context-aware transmission of messages by high-capacity (SBC) and constrained (ultra-low-power) devices to the cloud platform using publish-subscribe systems. We also point out the energy-efficiency importance for both devices and the cloud platform.

### Context-aware publish-subscribe messages

Publish-subscribe systems are payload-agnostic, and developers design topics and payload to meet the application needs. Nevertheless, IoT devices are diverse, ranging from high capacity to ultra-low-power. Hence, this diverse characteristic of devices poses challenges to designing a structure of topic and payload.

Considering the myriad of sensors and the absence of a standard, topic and payload range from tens to hundreds of bytes as observed in [19, 21].

Accordingly, an important discussion arises regarding standardization. First, the topic must identify the data source to the cloud platform process and store it. Cloud platforms, such as Azure 1 and AWS 2 present guidelines regarding the topic construction. However, these guidelines do not focus on the energy efficiency of the topic nor provide unique identification of devices. Using Azure as an example, we have a topic with 60 bytes. AWS, in turn, requires approximately 30 Bytes for telemetry data (topic can be greater according to the deviceID or other functions at the platform). Both platforms do not provide a standard for the unique identification of devices.

Besides, JSON is one of the main structures used when there is no standard to follow [35, 36]. Although, the JSON structure alone requires at least 7 bytes at the payload: {“”:“”} - without considering the sensor type used. About IoT sensors, the various types of sensors require efforts to identify them. For instance, the temperature sensor data, in a JSON payload can be: {“temperature”:“15.01”}. Also, a developer can use shorter metadata, like: {“t”:“15.01”}, but it might be a pitfall because for an IoT architecture with various domains and sensors, the character “t” can represent other sensors, not only temperature.

Thus, emerged the necessity to standardize the topic and the payload. As stated by [42], standardization decreases the gaps and reduces system complexity. Our LWPubSub message structure goes in this direction once we provide a unique topic for each device and standardized metadata on the payload.

### Cloud-connected devices

Considering the diverse characteristic of IoT devices presented in the sections “Introduction” and “Related work” and the necessity of them being able to connect to the Internet (and to the cloud), the devices that meet these requirements are: SBC and WSN (6LoWPAN) devices.

LPWAN devices (such as LoRa and SigFox) require a proxy that receives the message from the devices and forwards them to the cloud, breaking end-to-end communication (and security).

### Energy-efficiency

Energy efficiency is a crucial characteristic for the devices and the cloud platform. As presented by [43], the cloud computing metrics use the amount of data traffic to calculate the Key Performance Indicators (KPIs) related to energy efficiency and Greenhouse Gases (GHG) emissions.

Regarding corporate-level metrics, we observe some organizations using the metric *Carbon Intensity*, represented by the division of Scope 1 and 2 emissions by the total amount of data transported over its network (*CO*_2_/*Terabyte*) [43], as presented in Eq. 1.

1 $$  Carbon \; Intensity = \frac{GHG \; emissions \; Scope \; 1 \; and \; 2}{Terabytes \; of \; data \; traffic}  $$

The Power Usage Effectiveness (PUE) is a metric that shows progress in data center energy efficiency [44], according to Eq. 2, and it is widely used to observe data center efficiency, recommended by the GHG Protocol [44].

2 $$  PUE = \frac{Total \; Facility \; Power}{IT \; Equipment \; Power}  $$

Moreover, concerning the equipment-level metrics at the cloud platform, the Energy Consumption Rating Weighted (ECRW) represents the energy necessary to move *n* Gbps of data in Watt/Gbps [43], according to the Eq. 3.

3 $$  ECRW = \frac{((\alpha \times E_{f}) + (\beta \times E_{h}) + (\gamma \times E_{i}))}{T_{f}}  $$

where *T*_*f*_ is the maximum throughput (in Gbps); *E*_*f*_ is the energy consumption (in Watts) at maximum capacity; *E*_*h*_ is the energy consumption at half capacity; *E*_*i*_ is the energy consumption in idle; *α*=0.35,*β*=0.4, and *γ*=0.25 are the coefficients to represent the mixed mode of operation.

We observe in Eqs. 1, 2, and 3 that the number of bytes of the messages directly affect the energy consumption of the cloud platform. Thus, when constructing the topic and the payload, it is necessary to optimize the required bytes of a message.

Therefore, we conclude that reducing the number of bytes transmitted at each publish-subscribe message decreases the energy consumption, PUE, and Carbon Intensity of a cloud platform.

About devices, attention to the number of bytes remains equally important.

The energy consumption of IoT devices depends on the platform. High-capacity devices (with the 802.11 interface on) have the same energy consumption when performing sensing tasks (except when using CPU at 100% constantly - which is not the case of sensing data) [45]. So, each characteristic of the devices is considered to consume an amount of energy, for example, video output and Bluetooth, to calculate high capacity device’s energy consumption. Moreover, high-capacity devices used for remote sensing only require processing and transmission capability. Thus, devices must operate heedlessly.

On the other hand, ultra-low-power devices have more granularity to measure energy consumption. As presented by [46, 47] TX and RX are the resources that require more energy on these devices. Also, it is important to observe the CPU and Low Power Mode (LPM) energy consumption on them.

Moreover, for ultra-low-power devices, the application also needs to consider the 127-bytes MTU of 6LoWPAN devices.

### Message system

**Fig. 3:**
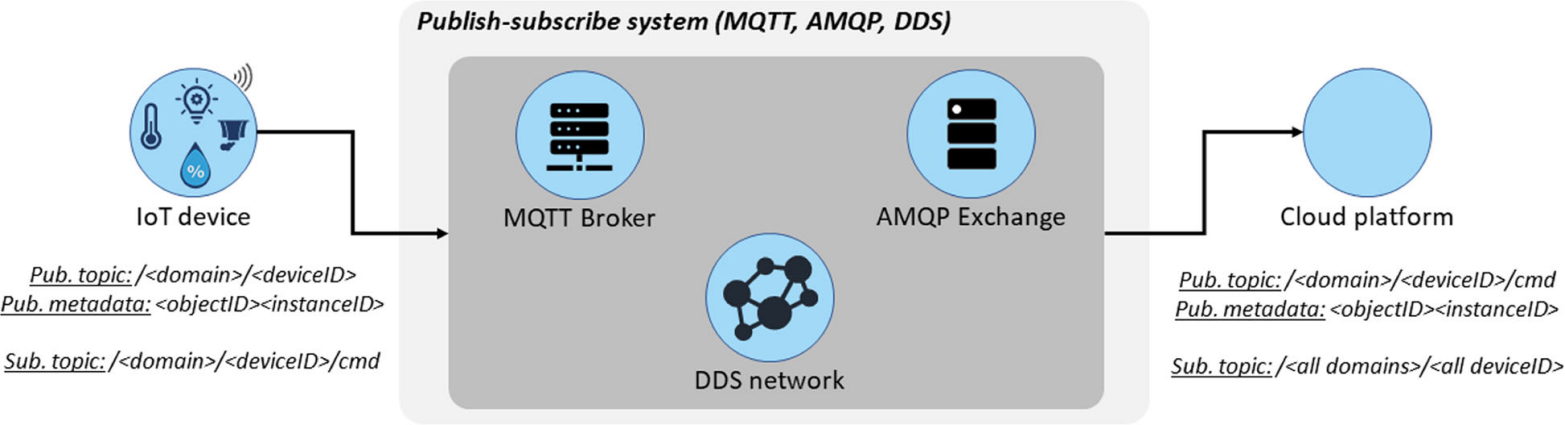
Structure of the LWPubSub message structure for publish-subscribe messages

Therefore, we present in Fig. 3 the proposed structure of topic and payload for publish-subscribe systems. The domains are the vertical markets [3] that enable the wide range of IoT architectures. A given IoT architecture, can contain the following domains: school, healthcare, transportation, among others (this work does not restrict the design of the domain, one can use a numeric sequence). Each domain has its own devices, uniquely identified by the *deviceID* metadata. Our proposal regarding *deviceID* is that the unique identification of the device must come from the MAC address. For that reason, the tuple /*domain*/*deviceID* uniquely identify a device at an IoT Architecture.

Regarding the sensors and actuators of a device, we propose the use of the IPSO registry [48], using the tuple *objectID*/*instanceID* to uniquely identify the devices’ sensors, where *objectID* is the type of the sensor and *instanceID* is the sensor itself.

The use of IPSO objects requires at most 5 bytes to identify a sensor, plus an additional byte to identify the instance of the sensor (in cases where a device has two or more sensors of the same type). For instance, assuming a device with two LEDs, IPSO defines the *objectID*3303, followed by the *instanceID*0 for the first and 1 for the other. Thus, compared to the JSON payload, the proposed use of IPSO objects meets the context-aware requirements while being energy-efficient.

With the union of /*domain*/*deviceID* (at the topic) and *objectIDinstanceID* (at the payload), we achieve lower byte usage for publish-subscribe systems.

When using MQTT, AMQP, or DDS publish-subscribe messages, the LWPubSub message structure sends the topic in plain text, which guarantees the end-to-end transmission of messages between clients without the need for an intermediate proxy.

Besides, the payload needs encryption to guarantee end-to-end security. Even using the standard best practices (TLS or DTLS), if the payload remains unencrypted, the Broker (MQTT/AMQP) or a subscriber (DDS) can read the message - resulting in data leakage. For this reason, LWPubSub applies confidentiality, authenticity, and integrity (CIA) to the payload.

### Security

Without security on the payload, an intermediary between the cloud and the devices (like the Broker) can read the messages, even when using TLS. Thus, the payload requires security. Applying confidentiality only, although possible, does not offer non-repudiation of the data transmitted. For this reason, it is crucial to use the CIA on the messages, providing Authenticated Encryption (AE). The LWPubSub message structure provides CIA to the publish-subscribe message payload using the Advanced Encryption Standard (AES) Counter Mode with Cipher Block Chaining Message Authentication Code (CCM) [25]. Besides, authenticated encryption provides security against chosen-ciphertext attacks.

The AES-CCM authenticates but does not encrypt the topic (the associated data), generating the authentication tag. The LWPubSub encrypts the payload, ensuring CIA. Further, AES-CCM requires a unique Nonce, and it provides a reduced message size and absence of padding.

The LWPubSub message structure encrypts and generates the payload according to these steps:

1. The first 13 bytes refer to the Nonce;
2. The following 8 bytes refer to the Message Integrity Code (MIC) - or authentication tag;
3. The subsequent bytes refer to the encrypted message.

About the IoT Architecture, we consider end-to-end secure the messages transmitted between the devices and the IoT Agent at the cloud platform.

## System evaluation and experiments

In this section, we introduce simulation scenarios, including parameter settings. We conducted several experiments to evaluate the proposed message system.

We observe and evaluate, with the execution of the experiments, the following metrics regarding the use of LWPubSub on MQTT, AMQP, and DDS:

- Header overhead;
- Energy consumption (observation of the behavior of devices in different operating conditions, obtaining measurements in poll frequency variations – various measurements by minute such as a wearable, or a few measurements by day as an environmental sensor);
- Number of bytes required for topic and payload.

**Fig. 4:**
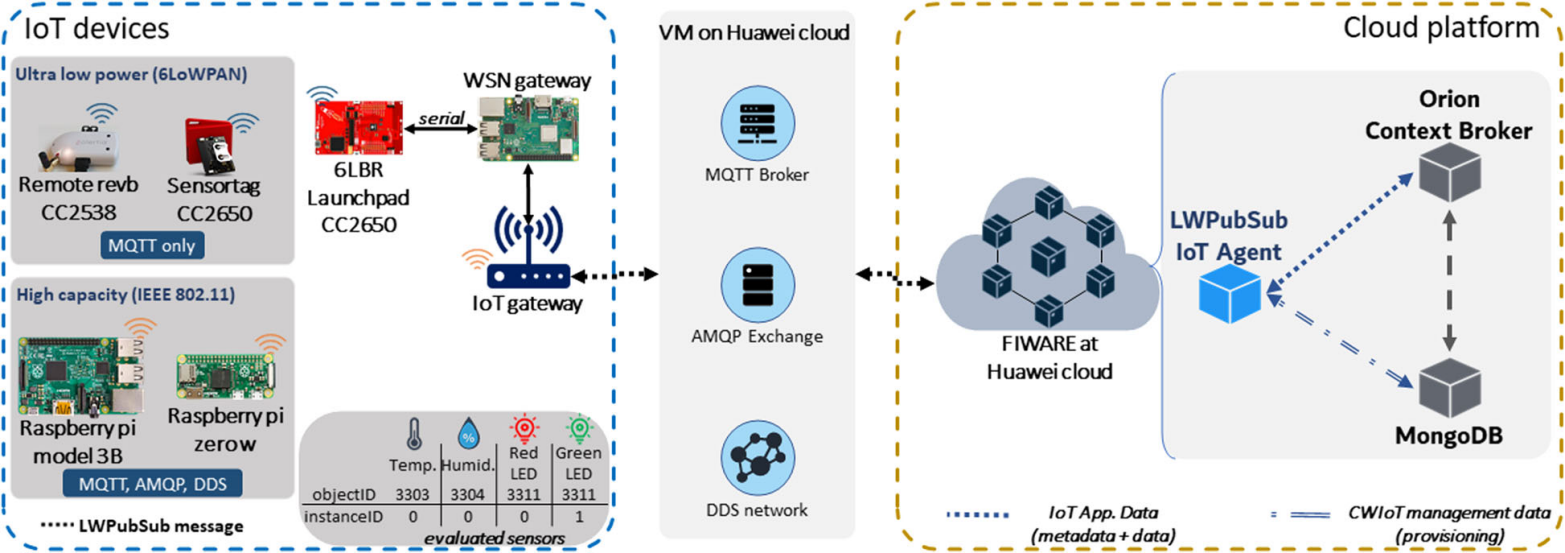
Experimental scenario

For the LWPubSub message structure validation and to collect the results, we use the experimental scenario presented in Fig 4.

In Table 3, we summarize the characteristics of the equipment, devices, sensors, and parameters used in our proposed message structure.

**Table 3:** Experiment parameters

**Equipment and Devices**		
Cloud platform	Microsservices/docker containers	
MQTT Broker	Mosquitto on the Huawei cloud	
AMQP Broker	RabbitMQ on the Huawei cloud	
DDS	OpenDDS on the Huawei cloud	
6LBR	Launchpad CC2650	
IoT Gateway	Raspberry pi model 3B	
Ultra-low-power devices	Sensortag CC2650	
	Remote CC2538	
High capacity devices	Raspberry Pi model 3B	
	Raspberry Pi Zero W	
**Cloud platform parameters**		
Platform	FIWARE-based	
Context-Broker	Orion 2.3.0	
Database	Mongo DB 3.6	
**ultra-low-power devices features**		
**Resource**	**Sensortag**	**Remote**
TX current	7.9 mA	24 mA
RX current	6 mA	20 mA
LPM current	0.55 mA	0.60 mA
CPU current	3.48 mA	20 mA
Microcontroller	CC2650 (48 MHz ARM Cortex M3)	CC2538 (32 MHz ARM Cortex M3)
ROM	128 KB	512 KB
RAM	20 KB	32 KB
OS	Contiki-NG	
TSCH schedule	Minimal	
**High capacity devices features**		
**Resource**	**Rasp. Pi model 3B**	**Rasp. Pi Zero W**
CPU	1.2 GHz 64-bit quad core ARM Cortex-A53	1 GHz ARM11 32-bit
Flash memory	32 GB	32 GB
RAM	1 GB	512 MB
802.11 active (headless)*	225.17 mA	95.15 mA
OS	Raspbian release 10	
**LWPubSub message structure parameters**		
Domain	99	
Sensortag deviceID	00124b05257a	
Remote deviceID	00124b4a527d	
Rasp. Pi 3B deviceID	0012eb00f6d0	
Rasp. Pi Zero W deviceID	0012ebc894cb	
*objectID*	3303, 3311, and 3338	
ultra-low-power devices security	Payload with AES-CCM-8	
High capacity devices security	TLS + payload with AES-CCM-8	

*measured with USB Voltmeter Tester UM24C

To validate the IoT architecture and considering as a premise the use of open standards, the FIWARE platform [13, 49, 50] provides the needed infrastructure for an opensource cloud. The FIWARE platform contains Generic Enablers (GE), including services called IoT Agents that receive IoT measurements. The Orion Context-Broker (the main GE) is the context-aware service provided by FIWARE. Contextualized data enable the correct processing of information by the cloud platform [49].

In the cloud platform, the domains and devices are orchestrated and provisioned, according to Table 3. Devices’ sensors are (following the LWPubSub structure *objectIDinstanceID*): 33030 (HDC temperature sensor), 33110 (red LED), 33111 (green LED), and 33380 (alarm).

The LWPubSub assembles the message (topic and payload) as shown in Fig. 3. An example of topic used in our experiments is (using Sensortag *deviceID* in this example) /99/00124b05257a, followed by the encrypted payload which contains a context-aware temperature data 33030|15.01.

The publish-subscribe systems, at the cloud platform, run at the IoT Agent on the Huawei cloud. The devices transmit their data to the cloud through the IoT gateway. The IoT gateway is a Raspberry Pi model 3B, with an IEEE 802.11 interface connected to the Internet and an IEEE 802.15.4 interface (via Launchpad, the 6LBR) to communicate with the 6LoWPAN network.

The ultra-low-power devices of the validation scenario are the Texas Instruments Sensortag and the Zolertia Remote rev. b. To comply with the energy-efficient goal of the LWPubSub, we use the Time Slotted Channel Hopping (TSCH) as the link-layer protocol since it presents the lowest energy consumption relating to this layer on 6LoWPAN networks [51] (we use TSCH minimal schedule). We use Contiki-NG, an open-source Operating System (OS) for constrained devices. As presented in section “Related work”, ultra-low-power devices can run only MQTT at the application layer.

In contrast, high-capacity devices run MQTT, AMQP, and DDS in the experiments.

Regardless of devices’ capacity, we implement AES-CCM-8 at the payload, ensuring end-to-end security between the devices and the cloud platform.

Moreover, IoT applications require different time intervals when collecting sensor measurements, ranging from wearable (which requires many measurements per minute) to environmental conditions measures (which require only a few measurements per day). Therefore, to observe the behavior of IoT in different operating conditions, the experiments conducted include the following poll frequencies to obtain and send measurements:

- Very high: one measurement every 5 seconds (17280 per day);
- High: one measurement every quarter of a minute (5760 per day);
- Medium: one measurement every quarter of an hour (96 per day);
- Low: one measurement every quarter of a day (4 per day);
- Very low: one measurement per day.

## Results and discussion

In this section, we analyze the impact of the publish-subscribe message systems considering several significant parameters and discuss the performance of the proposed message system through experiments.

### Publish-subscribe message size

About the topic, the LWPubSub message structure requires 16 bytes for sending measurements and 20 bytes to receive commands from the cloud platform. Concerning the payload presented in the section “System model”, to send telemetry data such as temperature or humidity, the secure payload is 32 bytes long. Thus, the entire LWPubSub message (topic+payload with end-to-end security) requires 52 bytes to send one-sensor data (temperature, for instance).

There are differences between the header overhead of the AMQP, DDS, and MQTT protocols when using LWPubSub. We present the overhead based on the required bytes in the header and the total message size. Thereupon, we need to observe the header overhead percentage, which we calculate using the Eq. 4, considering the header size presented in the section “Related work”.

4 $$  {}Header \; overhead (\%) = \frac{Header}{Header+Payload} \times 100  $$

**Fig. 5:**
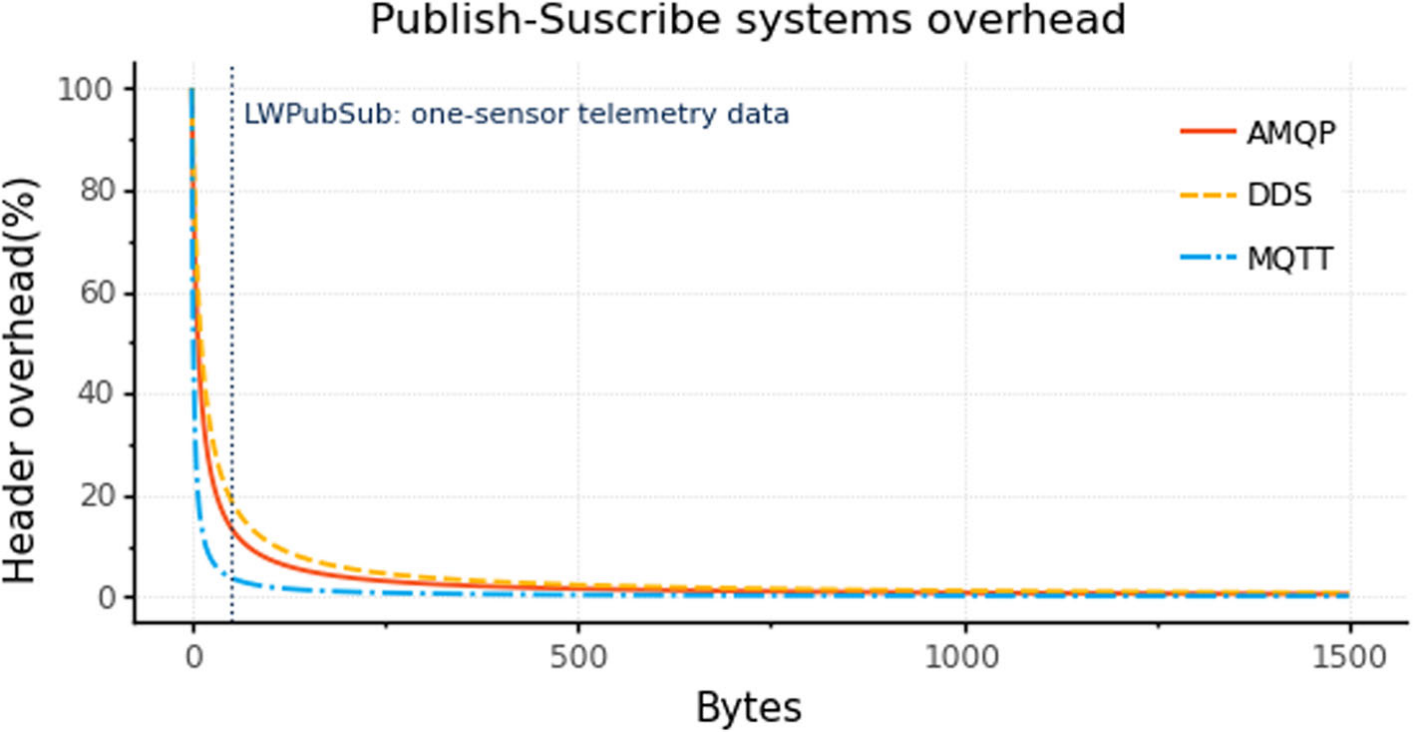
Header overhead using LWPubSub messages

In Fig. 5 we observe the header overhead required by AMQP, DDS, and MQTT when using LWPubSub topic and payload. The dashed line in Fig. 5 represents the header overhead with the use of LWPubSub to send temperature data. MQTT presents the lower header overhead (3.70%), followed by AMQP (13.33%) and DDS (18.75%). It is important to highlight the relevance of the protocol overhead, given the constrained payload of ultra-low-power devices (127 bytes), where only 104 bytes are available. Thus, above 104 bytes, the message requires fragmentation, increasing the number of messages to send the data and energy consumption.

Regarding SBC, which does not have payload-size restrictions, the header overhead is not significant for larger messages. For example, the header overhead of a payload of 1185 bytes is below 1% for MQTT, AMQP, and DDS.

The structure design of our message proposes a short and lightweight (and yet, complete) topic. We assume the cloud platform as the main entity that publicly provides the IoT data to the users. The publish-subscribe system (MQTT, AMQP, DDS) is only accessible between the devices and the cloud platform and not by other users.

**Fig. 6:**
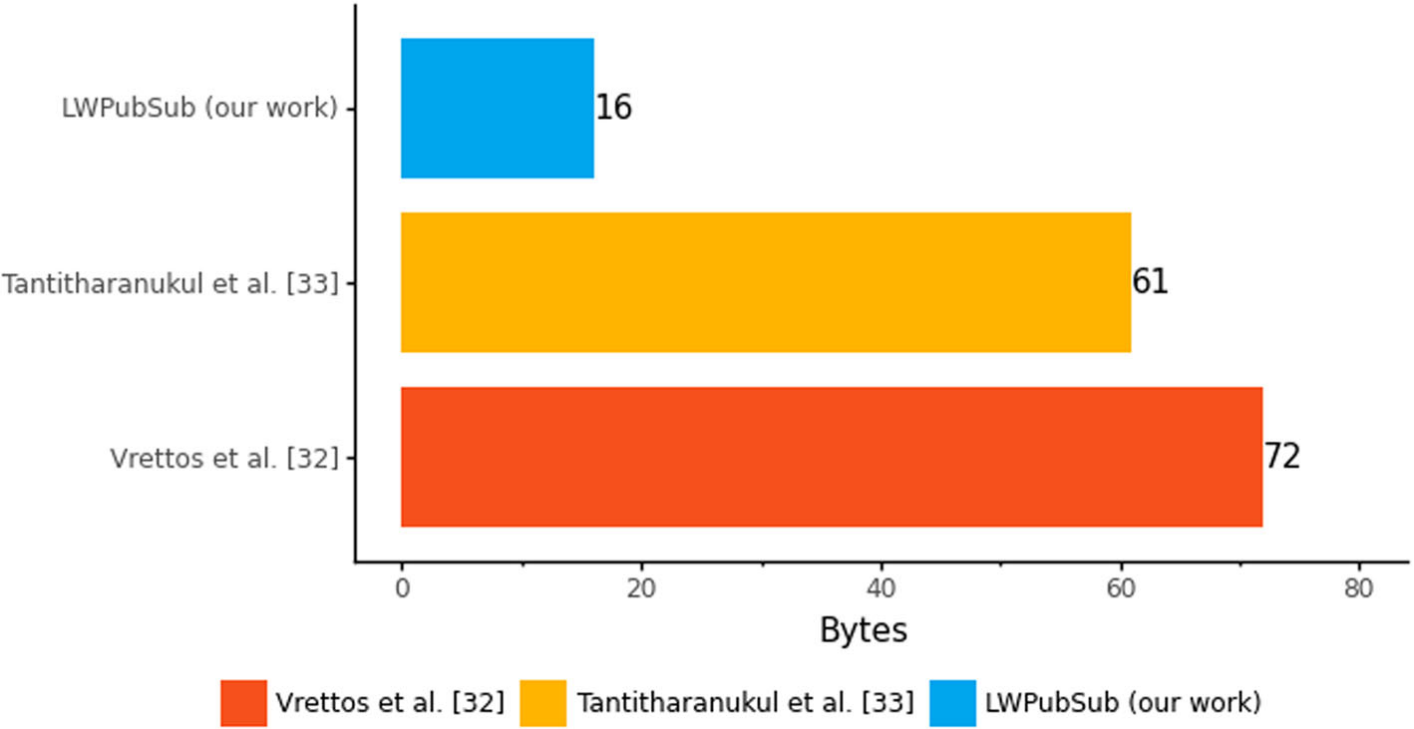
Topic results comparison with the main related works

In contrast, [33] proposes a topic naming criteria for smart cities requiring users’ access to the Broker. The topic structure proposed by [33] requires, at least, the fields “Objective/Location/Owner” and its sub-fields. For instance, in the example provided by the authors, a temperature measurement topic requires 61 bytes. The topic proposed by [32] requires 72 bytes - we consider “country” and “state” fields with 2 bytes to reduce the number of bytes on the topic. On the other hand, our proposed topic for sending temperature requires 16 bytes. Figure 6 presents the topic comparison with the main related works.

In the works of [32, 33], the client needs to know the Broker’s address and to subscribe to the desired topics. Our work, in turn, proposes cloud computing as the primary entity which provides the IoT data to the users.

Regarding the ultra-low-power devices (which use only MQTT), to send one-sensor telemetry data, the LWPubSub message (with CIA) generates a 6LoWPAN packet of 113 bytes in the experiments. Thus, there is no fragmentation of messages sent by the ultra-low-power devices (which have a messaging limit of 127 bytes). For high-capacity devices, the payload is 106-bytes long.

**Fig. 7:**
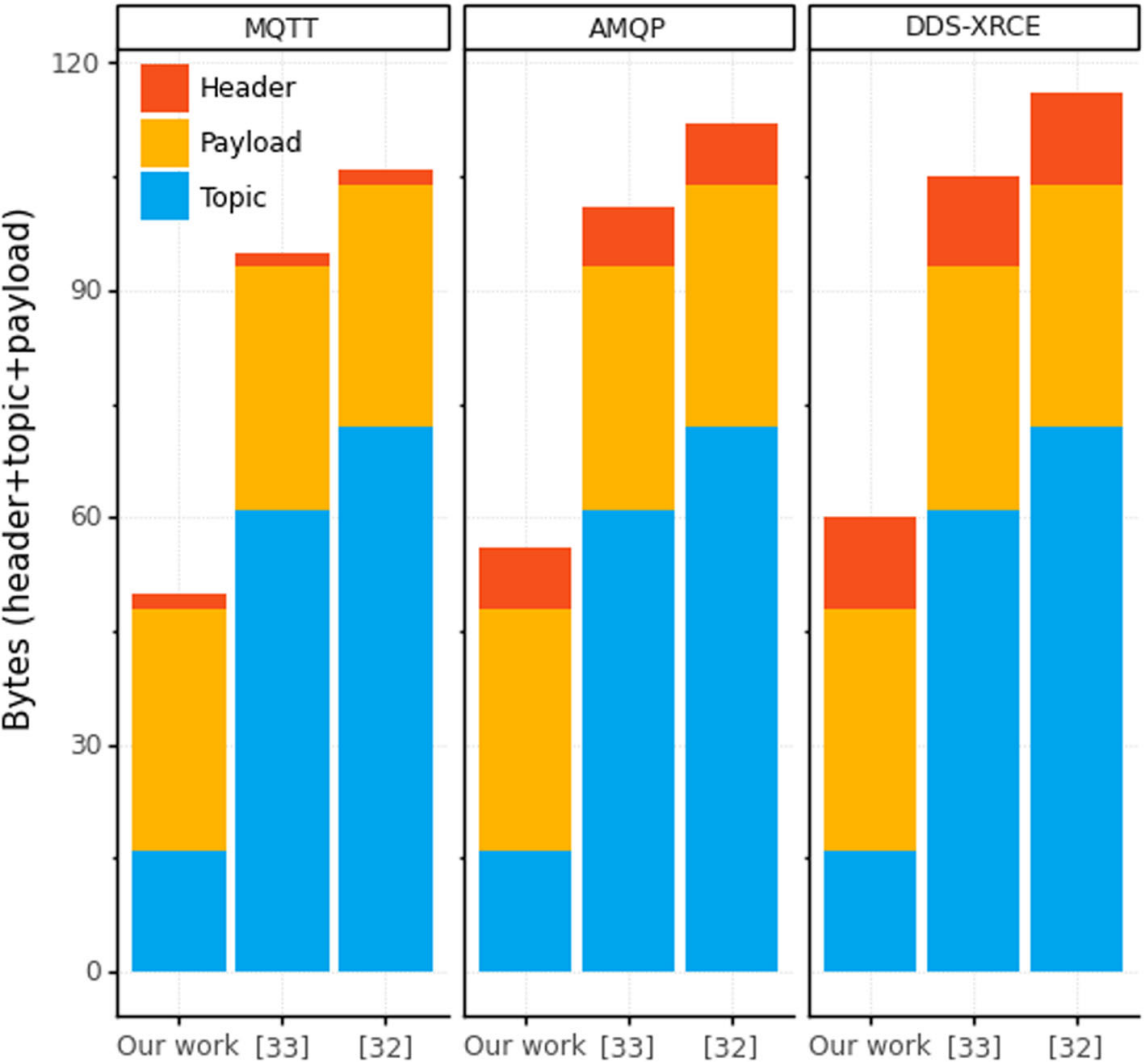
Message size comparison with the main related works

We present in Fig. 7 the comparison of the message size among the main related works. We consider our context-aware and secure payload (32 bytes) for all works compared; however, each work has its respective topic. The LWPubSub message presents a smaller message size, thus, requiring the transmission of fewer bytes and saving energy.

### Energy consumption

On the subject of energy consumption, we apply the Eq. 5 to observe the energy consumption considering idle and active periods of the IoT devices; thus, the parameter *n* varies depending on the type of IoT device used.

5 $$  Energy_{IoT device} = \sum_{i=1}^{n} n \times V \times I  $$

In Eq. 5, *n* is the time spent for device resource, *V* is the voltage, and *I* is the current. Regarding high capacity devices, we deploy a headless Raspberry Pi model 3B and Raspberry Pi Zero W. We measure the energy consumption with the devices in idle and active (transmission and reception using the 802.11 radio). According to Eq. 5, the energy consumption presents the time spent considering *V*=5, the current (I) as presented in Table 3, and *n*=(*Idle, TX/RX*).

About the energy consumption calculation of 6LoWPAN devices (ultra-low-power), we proceed according to Eq. 5, using the Contiki-NG Energest [52] module. We calculate the energy (in millijoules) from the time spent (in seconds) of each device resource (CPU, LPM, TX, RX). The voltage is fixed at 3 V (when supplied by two AA batteries), and the current (I) for each resource is shown in Table 3, and *n*=(*CPU, LPM, TX, RX*).

The SBC devices evaluated, as presented in Fig. 4, are capable of running MQTT, AMQP, and DDS. However, the ultra-low-power devices are only capable of running MQTT, given their constrained nature.

**Fig. 8:**
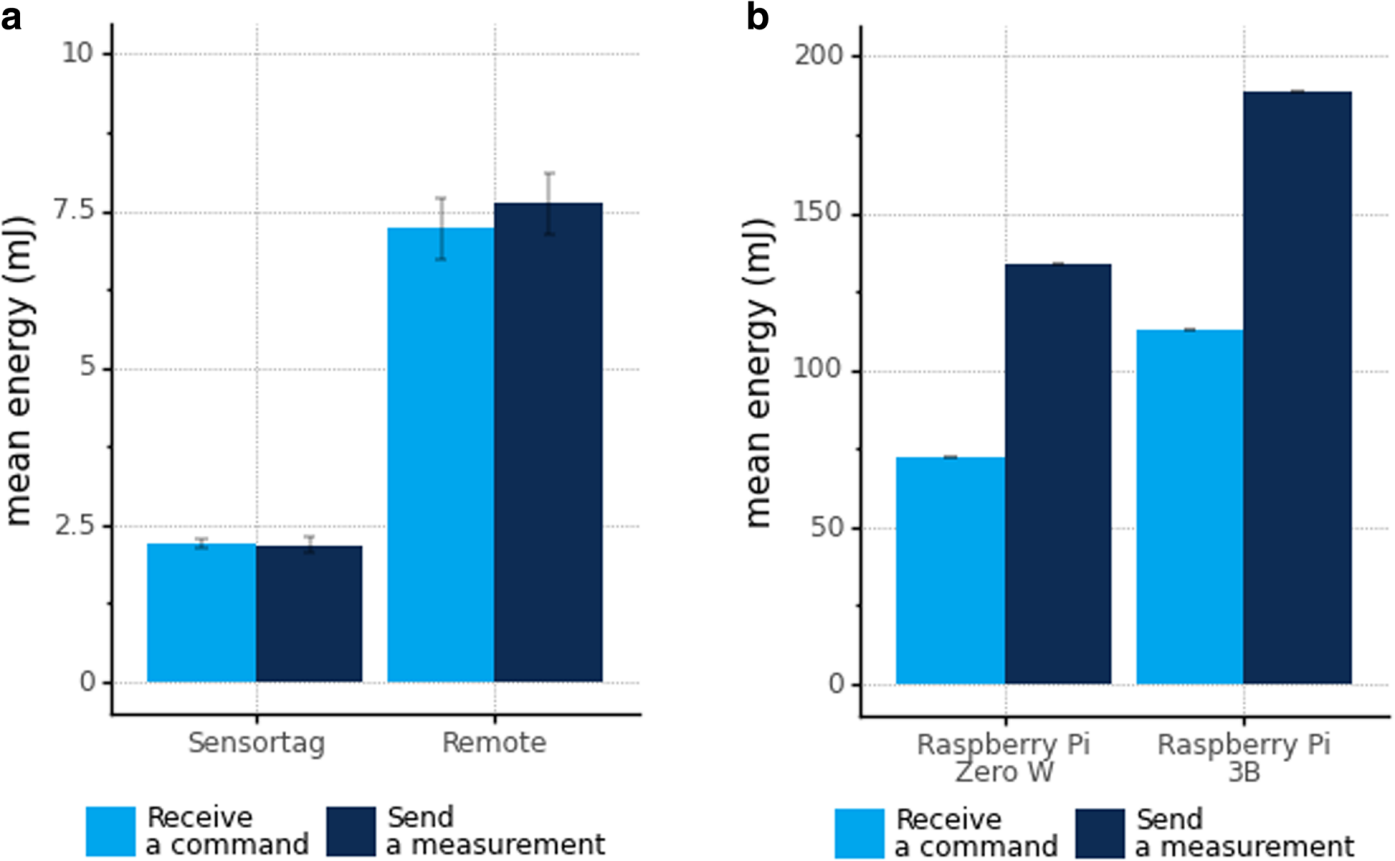
Energy consumption to send measurements and receive commands

We present in Fig. 8 the energy consumption for an IoT device to send and receive one-sensor LWPubSub messages. It is important to note the y-axis limits because, for ultra-low-power devices, the energy required is under 10mJ; nevertheless, SBC devices require much more energy, reaching almost 200 mJ. Moreover, in Fig. 8, we consider the actions “send a measurement” and “receive a command”, not considering the idle time.

**Fig. 9:**
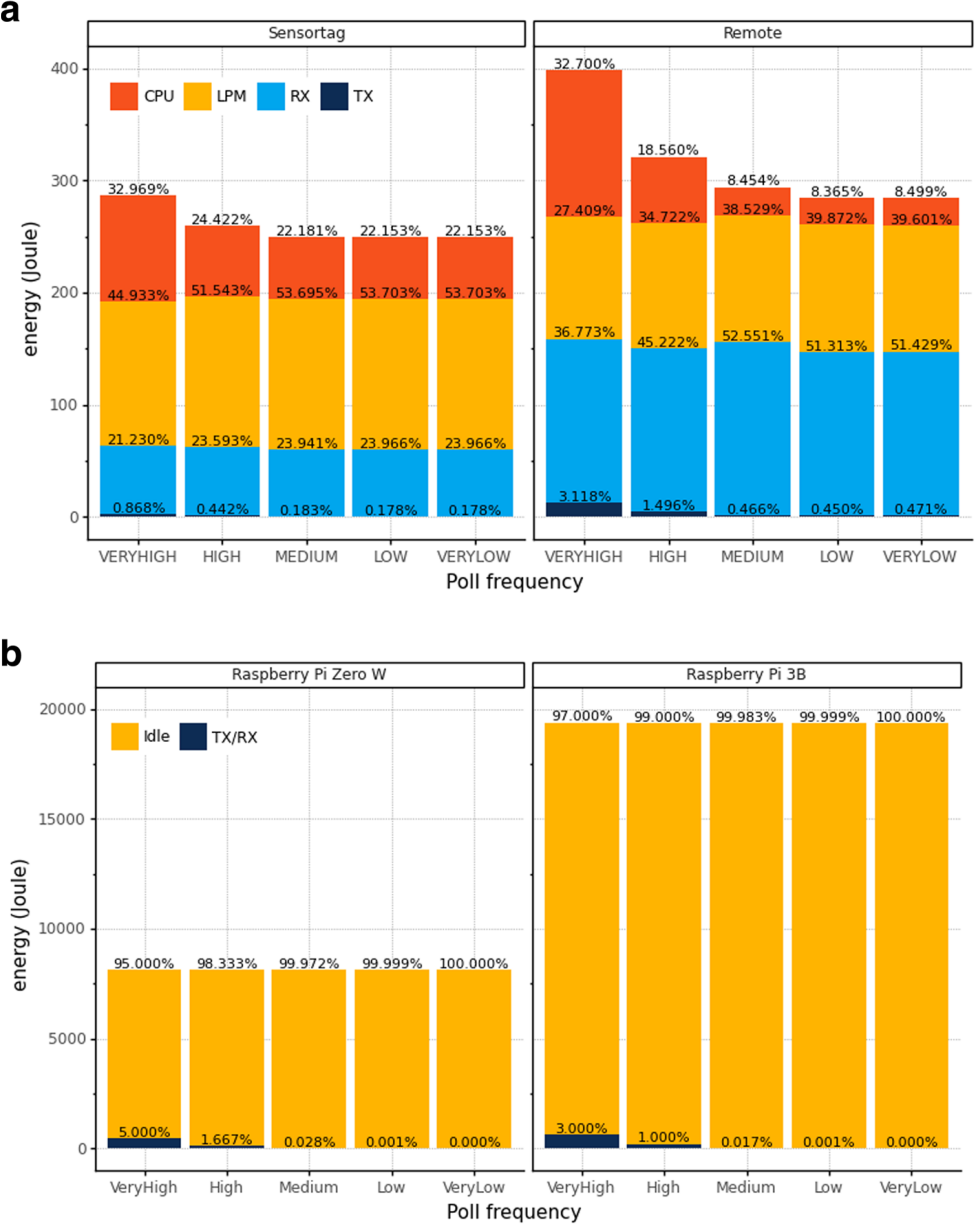
Daily energy consumption of IoT devices

Additionally, Fig. 9 represents the comparison of the required daily energy of ultra-low-power and SBC devices.

Besides, Fig. 9a shows the ultra-low-power devices consuming less than 400 J (Sensortag is more constrained and consumes less than 300 J). We observe that sleep time (LPM) and listening (RX) drive ultra-low-power devices’ energy consumption. The CPU and TX energy consumption is almost the same amount for medium, low, and very low poll frequencies.

Regarding the energy consumption of SBC presented in Fig. 9b, it reaches approximately 8,000 J for Raspberry Pi Zero W and almost 20,000 J for the Raspberry Pi 3B, regardless of the publish-subscribe message system (MQTT, AMQP, or DDS). SBC spends most of its time in “idle”, even for the poll frequency “very high”.

We observe the previous comparison of energy consumption when looking at the battery life of the devices at each poll frequency. Let us consider the energy provided by two AA batteries: 30780 J (2.85*Ah*∗1.5*V*∗3600*secs*.∗2).

**Fig. 10:**
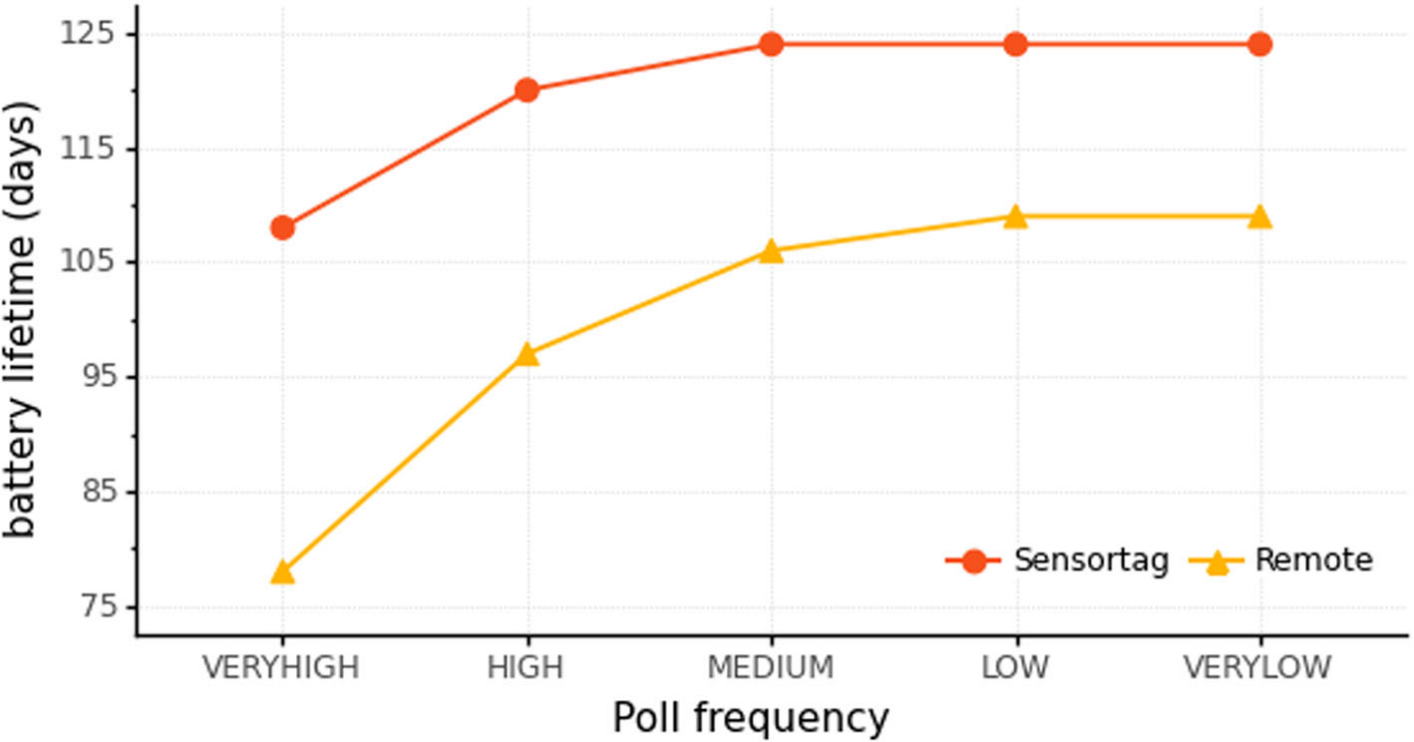
Ultra-low-power devices battery lifetime on the evaluated poll frequencies

With this energy, the Raspberry Pi 3B and Raspberry Pi Zero W run for 1.5 and 3.7 days, respectively, regardless of the poll frequency. In contrast, ultra-low-power devices present a different behavior. Figure 10 illustrates the battery lifetime of ultra-low-power devices at the evaluated poll frequencies. The “very high” and “high” poll frequencies presented in Fig. 10 (which demand more energy) present lower battery life than the other poll frequencies. However, sending 96, 4, or 1 message per day (medium, low, and very low poll frequencies) consumes nearly the same amount of energy - even for those that wait longer between measurements such as “low” and “very low”.

Thus, high-capacity devices must have an uninterruptible power supply; in contrast, ultra-low-power devices can run on a battery for months, enabling mobility.

Regarding security, for an IoT application that requires the best security practices, SBC is the device to use. On the other hand, TLS is not an option for ultra-low-power devices once they cannot run this heavy-weight security protocol. However, as presented in the section “System model”, the LWPubSub message structure applies confidentiality, integrity, and authenticity to the payload.

### IoT applications

A smart healthcare IoT architecture can be benefited from the use of ultra-low-power devices using LWPubSub. A tracking system for vaccine temperature control is an example of an application. It is possible to measure the temperature of a vaccine box from the production to the destination once the LWPubSub application periodically publishes these data. Besides, these devices can join 6LoWPAN networks when in their range; in contrast, an SBC device (which uses the 802.11 radio) requires a previous wireless network configuration to transmit data. If there is a 6LoWPAN network in the industry, at the transportation, and the destination, it is possible to track the vaccine box temperature end-to-end because the device joins the respective network and keeps sending the data. In addition, making the measured temperature publicly available to citizens and the scientific community increases the transparency of information, especially with respect to the Covid-19 pandemic.

Healthcare for the elderly is another domain of smart healthcare that can be benefited from the ultra-low-power devices using LWPubSub. Even collecting data at a higher poll frequency, ultra-low-power devices run months without requiring battery replacement.

High-capacity devices are helpful to send a large amount of data, for instance, a security camera that detects an anomaly and sends the images to the cloud so that authorities can analyze them.

These applications, using LWPubSub, transmit context-aware and end-to-end secure messages between the IoT devices and the cloud platform, and the Broker (or any intermediary subscriber on DDS networks) is unable to read the message.

## Conclusion

This paper proposes and evaluates an energy-efficient, context-aware, and end-to-end secure message structure for publish-subscribe systems. Both ultra-low-power and high-capacity devices benefit from the optimized topic and payload proposed.

The context-aware topic guarantees the unique device identification on its domain. The context-aware payload ensures the standardization of each device’s sensors, including end-to-end security between the devices and the cloud platform.

We highlight the importance of the reduced number of bytes at the topic and the payload of messages to reduce energy consumption because carbon footprint and power effectiveness metrics are based on the number of bytes transmitted.

The proposed message system does not require customizing the protocols (MQTT, AMQP, or DDS) or the Broker. Thus, it is possible to use any Broker available to forward the secure messages to the cloud platform.

Experimental evaluation shows that our message system can run for months on ultra-low-power devices. We also present IoT applications that can benefit from the message system. To achieve this contribution, we execute several experiments applying different poll frequencies to evaluate the diverse characteristics of IoT applications.

**Limitations and Future works**

Although the results obtained prove the low energy consumption and the message size without resorting to fragmentation for ultra-low-power devices, the evaluation comprises one measurement from one sensor at a time. An extension of this work could be evaluating the message size to verify if sending measurements from more than one sensor in the same message requires fragmentation, its impact on energy consumption, and other implications this may cause. Additionally, for ultra-low-power devices, explore other encryption models that can be adopted when considering security, using key derivation functions and features to deliver the static keys, requiring analysis of the message size and energy consumption. Regarding total energy consumption, future works may include evaluating another TSCH Schedule.

## Data Availability

The test cases are generated by an algorithm, which will be made available under an appropriate open source license upon accepting the article.

## Footnotes

1 https://docs.microsoft.com/en-us/azure/iot-hub/iot-hub-mqtt-support

2 https://docs.aws.amazon.com/whitepapers/latest/designing-mqtt-topics-aws-iot-core/designing-mqtt-topics-aws-iot-core.html
